# Staying connected during the COVID-19 pandemic

**DOI:** 10.1177/0020764020926562

**Published:** 2020-05-08

**Authors:** Qin Xiang Ng, Kuan Tsee Chee, Michelle Lee Zhi Qing De Deyn, Zenn Chua

**Affiliations:** 1 Department of General and Community Psychiatry, Institute of Mental Health, Buangkok Green Medical Park, Singapore; 2 MOH Holdings Pte Ltd., Singapore; 3 Department of Medicine, James Cook University Hospital, Middlesbrough, UK

We read with great interest the publication by Torales et al. (2020) and thank his team for highlighting the potential adverse impact of the Coronavirus Disease 2019 (COVID-19) on global mental health. In the wake of the COVID-19 pandemic, which has now infected more than two million people worldwide, strict quarantine measures are a commonplace and a third of the world’s population have now gone into some form of lockdown (Business Insider Singapore, 2020). This includes some states in the United States, the United Kingdom, India, France, Germany, Italy, Spain, China and Singapore as well. Till date, it appears that the most effective strategies to slow the transmission of severe acute respiratory syndrome coronavirus 2 (SARS-CoV-2) are good hand hygiene, social distancing and physical quarantine for suspected or confirmed cases (Cochrane Library, 2020).

The adage that ‘no man is an island’ espouses the fundamental idea that human beings do badly when isolated from others and they need to be part of a community to thrive. There is overwhelming research to suggest that social isolation and loneliness are serious public health issues, especially among the elderly (Gerst-Emerson & Jayawardhana, 2015), and they are linked to increased mortality and morbidity (Holt-Lunstad et al., 2010). In a 2010 meta-analysis of social relationships and mortality risk in over 300,000 participants, it was found that the influence of social relationships on risk for mortality (odds ratio 1.50, 95% confidence interval 1.42–1.59) was comparable to well-established risk factors for mortality, for example, excessive alcohol consumption and smoking (Holt-Lunstad et al., 2010).

In our own clinical experience, anecdotally, we have seen increased numbers of patients with obsessive-compulsive disorder (OCD) or personality difficulties seeking psychiatric help in the recent months. Their conditions may be exacerbated by the fear of contagion and of loved ones falling ill or feelings of emptiness when quarantined from others. This may be especially true for the COVID-19 as we now live in a digital age and are constantly bombarded with information, misinformation or ‘fake news’. There is still much uncertainty surrounding the epidemiology and virology of the SARS-CoV-2, including the presence of asymptomatic spreaders and the variable incubation period for clinical disease (Lauer et al., 2020). There is also no definitive treatment or vaccine at the present moment.

To maximize existing resources, promote good mental health at home and improve access to mental health services for the population, we should leverage on telemental health services, including the use of psychiatric teleconsultation, videoconferencing and telehealth to deliver cognitive behavioural therapy (CBT) (Dent et al., 2018). Social support networks and establishing online support groups could also help people to stay connected during this pandemic. Although there are pilot studies to support the feasibility of psychiatric teleconsultations (Wichman et al., 2019) and most stable mental health patients would be appropriate candidates, the actual implementation and utilization rates of remote services, however, remain low globally due to legal uncertainty (Raposo, 2016), patient and physician unfamiliarity, as well as technical difficulties. The elderly may lack access and digital literacy is a significant barrier.

To safeguard against the vulnerable falling through the cracks, social befriending services should continue with regular (weekly) telephone calls and visit those uncontactable via phone and assessed as moderate risk and above. In our continued fight against the COVID-19 pandemic, we must not forget about its potential impact on mental health, especially among those with pre-existing psychiatric conditions, the elderly, the disadvantaged and the marginalized.
